# Influence of Different Aqueous Media on the Corrosion Behavior of B_4_C-Modified Lightweight Al-Mg-Si Matrix Composites

**DOI:** 10.3390/ma15238531

**Published:** 2022-11-30

**Authors:** Neeraj Kumar, Ashutosh Sharma, Manoranjan Kumar Manoj

**Affiliations:** 1 Metallurgical and Materials Engineering, National Institute of Technology (NIT), Raipur 492010, India; 2 Department of Metallurgical Engineering, School of Engineering (SOE), OP Jindal University (OPJU), Raigarh 496001, India; 3 Department of Materials Science and Engineering, Ajou University, Suwon 16499, Republic of Korea

**Keywords:** corrosion, powder metallurgy, metal matrix composite (MMC), ceramics, aluminum alloys

## Abstract

In this study, we have investigated the electrochemical corrosion behavior of boron carbide (B_4_C) ceramic-reinforced Al-Mg-Si matrix composites in various aqueous environments (NaOH, NaCl, HCl, and H_2_SO_4_). The samples were produced by the powder metallurgy (P/M) route and the corrosion investigations were conducted by potentiodynamic polarization (PDP) and electrochemical impedance spectroscopy (EIS) methods. The morphology of the as-prepared and corroded samples was examined by scanning electron microscopy (SEM) and energy dispersive spectroscopy (EDS) studies. The investigations revealed that the corrosion resistance of Al-Mg-Si composites is highest in NaCl medium due to a less negative corrosion potential, higher charge transfer (R_ct_) resistance, and lower double-layer capacitance (C_dl_) as compared to other media. The SEM morphology suggests that B_4_C ceramics enhance corrosion resistance by forming a protective barrier layer of OH- and Cl- deposits in the composite and unreinforced alloy, respectively.

## 1. Introduction

In recent years, the use of metal matrix composites (MMCs) has become more popular in the automobile, aerospace, and nuclear sectors. Among these, aluminum matrix composites (AMCs) are highly valued because of their vast array of applications in the automotive sector, such as brake drum, brake rotor, high-speed shaft, and engine block. AMCs possess good strength and are lightweight, factors directly linked to their efficiency, performance, and fuel consumption [1]. Most AMCs consist of common reinforcements, including SiC [2], MOSi_2_ [3], TiB_2_ [4], and TiO_2_ [5]. However, B_4_C appears to be efficient for AMCs owing to its high hardness, high elastic modulus, low density, high melting point, and excellent chemical stability [6]. The AMCs have been made possible with the aid of recent technologies, such as die-casting [7], sand casting [8], P/M route [9], friction stir processing [10], and stir casting [11]. Among these, P/M processing is the most effective process that allows strong interfacial bonding and provides a uniform dispersion of particles in the matrix alloy [12]. Although plenty of studies have been conducted on the mechanical [12,13] and tribological [14,15] properties of AMCs, relatively few systematic studies have been performed on the environmental corrosion of aluminum composites [9,16]. Hihara et al. [17] found that the galvanic corrosion current density of the composite with reinforcements SiC was of the order of 10^−4^ A/cm^2^. Besides, the matrix alloy showed more passivation behavior than the galvanic composites with scan rate = 0.1 mV/s in the Na_2_SO_4_ solution. In 0.5 M Na_2_SO_4_ with NaF, Hihara et al. [18] investigated the galvanic corrosion of SiC monofilament (MF) coupled to pure Mg alloy. When a small amount of NaF (5 g L^−1^) is added to electrolytes, it passivates metal matrix composites. Murthy et al. [19] reported that AMCs show lower corrosion resistance and increases of reinforcement in the Na_2_SO_4_ and Na_2_NO_3_ solutions. The comparison from both the media depicted that the relative degree of corrosion rate followed the order NO_3_ > SO_4_. The lowest rate of corrosion is due to the formation of passive layer over composites. Hihara et al. [20] observed that Mg MMCs showed lower corrosion current (3 × 10^−4^ A/cm^2^) in Na_2_SO_2_ as compared to deaerated 0.5 M Na_2_NO_3_ ((4 × 10^−4^ A/cm^2^). Recently, Ding et al. [21] investigated a semiconductor model for examining the enhanced cathodic currents of B_4_C under illumination in Al/B_4_C MMC in 0.5 M Na_2_SO_4_. They also detect the depletion layer at B4C/electrolytes interface by using a scanning vibrating electrode technique and scanning ion-selective electrode technique. Tiwari et al. [22] developed a corrosion model for AMCs used for transmission lines against weather and atmospheric conditions of Hawaii (H) and Illinois (IL). They found that the higher corrosion rate in marine (KH) than KIL environment (KH > KIL) can be attributed to the lower chloride amount. It has been noticed that as the volume fraction of the B_4_C increases, the corrosion behavior of the AMCs can be understood by the pitting and galvanic corrosion in the AMCs [23].

Recently, Mohammed and his colleagues [24] investigated the corrosion behavior of Al/B_4_C AMCs using PDP and EIS in four media and observed that the corrosion rate of AMCs is approximately 99% lower in the NaCl medium and 22–42% lower in NaOH and their charge transfer resistance is approximately 23–59% higher than the matrix alloy. According to the analysis published by Zeng et al. [25], the optimal condition for better corrosion resistance of Al/B_4_C MMCs was obtained at 25 wt% H_2_SO_4_, 25 V, and 12 °C. The corrosion resistance of AMCs is improved due to the dense and compact films over the composite surface. Furthermore, Hongho et al. [26] used a semiconductor model for studying the corrosion behavior of Al/B_4_C. Their model explained that galvanic couples form in AMCs at anodic and cathodic sites in around 200 microns. The same authors have also reported that localized corrosion occurs due to the acidified conditions of the anodic site, and therefore more alkaline conditions emerge on the cathodic sites of the reinforcement (B_4_C, SiC, and Al_2_O_3_) AMCs, in the presence of 0.5 M Na_2_SO_4_ [27].

Recent research has concentrated on the engineering and design of AMCs for achieving extraordinary mechanical and physical properties. However, there is no awareness or consideration taken for the deterioration of metal composites from the processing stage to the final product. How will they behave in a corrosive environment? Secondly, maximum aluminum composites have been designed and fabricated by ceramic particles through a liquid casting route, e.g., stir casting. During the synthesis process, the temperature of the stir reaches more than 800 °C, and at that time, matrix alloys start to nucleate new intermetallic phases/interfacial reaction products in the vicinity of composites, which provides a reverse impact and acceleration dissolution of matrix alloys, or in a nutshell, it acts as oxygen reducers, and aluminum goes for the high rate of dissolutions. As a result, the pH has also changed with respect to the matrix. This causes the matrix alloys to disintegrate all around the preferential cathode [27]. Keeping these concepts in mind, this issue can be minimized by using the novel P/M route. Further, it has been observed that relatively little information appears to be associated with the corrosion of metal matrix composites.

More notably, the present study aims to investigate the corrosion analysis by PDP and EIS methods, and to conduct a further evaluation of the microstructure and corrosion mechanism of B_4_C/Al-Mg-Si AMCs in aggressive environments (NaOH, NaCl, H_2_SO_4_, and HCl). Hardness and density measurements were also performed to correlate the changes in the microstructure of AMCs. The objective is to predict the suitable environments for automotive Al and AMCs components in prolonged service.

## 2. Materials and Methods

### 2.1. Materials and Fabrication of B_4_C/Al-Mg-Si AMCs Composites

**Figure 1:**
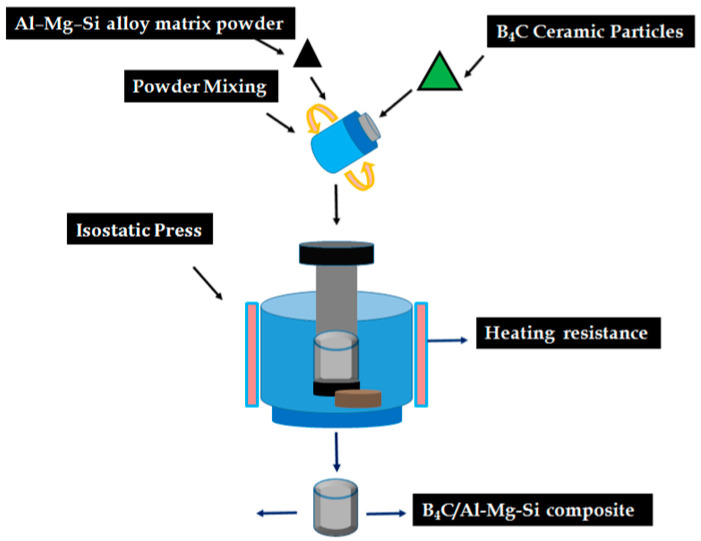
The schematic diagram for composite fabrication by P/M route in this study.

The AMCs included 17.5% of B_4_C and Al-Mg-Si alloy prepared through the P/M route. The particle size range of the B_4_C material was 10–34 µm, and the powder of the matrix alloy was 5–20 µm. After being cleaned with alcohol, the B_4_C reinforced particles were heated up in an oven at 400 °C for 1 h. Consequently, the B_4_C powder particles were mixed with the matrix alloy at 200 rpm and 6:1 ball to powder weight ratio under Ar environment, as shown in Figure 1. Ball milling was performed for a couple of hours using the ball milling machine (Retsch, PM-400, Haan, Germany) in the presence of toluene as a process control agent. Further, the milled powder mixture was isostatically cold-pressed in cylindrical dies at 50 MPa pressure and 15 min time to form pellets of Ø10 mm and 10 mm height. The cylindrical pellets, composite, as well as unreinforced alloy were sintered at 560 °C for 90 min under Ar environment in a tube furnace [28,29].

### 2.2. Electrochemical Corrosion Characterization

The corrosion studies were performed in four types of environments (NaOH, NaCl, HCl, H_2_SO_4_). All the chemicals were of reagent grade (>99.5% purity) obtained from Sigma Aldrich, USA. The composition and solution chemistry of the corrosive media are given in Table 1. The corrosion measurements were performed by using a three-electrode cell and Autolab PGSTAT 302N instrument. The electrodes included a platinum wire (Pt) and Ag/AgCl (3 M KCl) reference electrode while the sample served as an anode. Prior to investigations, the samples were polished metallographically and an area of 1.0 cm^2^ was exposed in the corrosion media for testing.

The open-circuit potential (OCP) was observed for 1 h to stabilize the potential window of the samples in the various electrolytes. PDP plots were recorded from −0.2 to −1.8 V vs. OCP at a scan rate of 0.1 mV/s. EIS experiments were conducted by applying an AC voltage of 5 mV amplitude over the frequency regime of 10 Hz–10,000 kHz.

### 2.3. Materials Characterization

The morphology of the as-prepared and corroded samples AMCs was examined. The samples were cold mounted in an epoxy resin and ground using SiC papers up to 2000 grit accompanied by washing with acetone, ethanol, and deionized water. The samples were then used as Keller’s reagents (95 mL water, 2.5 mL HNO_3_, 1.5 mL HCl, 1.0 mL HF) to observe the surface morphologies. The morphology of the powder particles and composite B_4_C/Al-Mg-Si AMCs were studied using scanning electron microscopy (SEM, Hitachi 4800S, Japan) coupled with energy-dispersive X-ray spectroscopy (EDS). The experimental densities of AMCs and matrix alloy were calculated by the Archimedes principle using an analytical balance (Shimadzu-AUX-220, SMK-401).

## 3. Results

### 3.1. Composite Fabrication and Morphology

**Figure 2:**
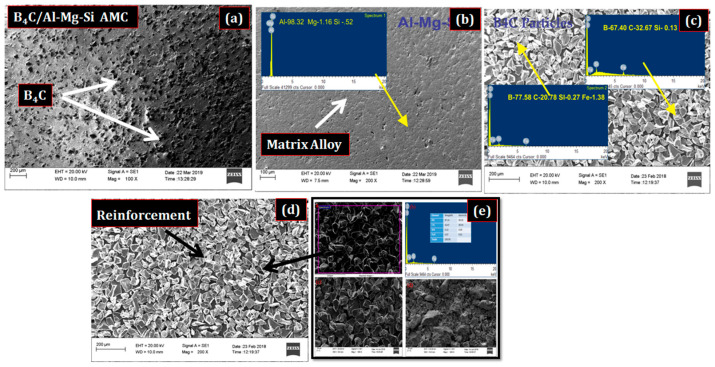
SEM images showing the composite morphologies. (**a**) 17.5% B_4_C/Al-Mg-Si AMCs (**b**) Al-Mg-Si matrix alloy, (**c**,**d**) B_4_C reinforcements, and (**e**) EDS of (**d**).

Figure 2a–e illustrates the morphology of B_4_C ceramic particles distributed in Al-Mg-Si alloy matrix. The SEM acquired morphology supports uniformly distributed and properly embedded particles without any void at the interface, as shown in Figure 2a.

The surface is well dispersed with the B_4_C reinforcements as compared the matrix alloy (Figure 2b). This can be attributed to the centrifugal influence that occurs evenly among the particles during the proper milling, as well as the further sintering process, which enables their inter-particle bonding in the composite [30,31,32,33,34,35]. The morphology and the presence of B_4_C particles in the matrix alloy were further confirmed by the EDS analysis, as shown in Figure 2c–e. Some unevenness can be observed in the matrix, which can be ascribed to the air entrapped inside the composite.

### 3.2. Hardness and Density Measurements

The measurement of Vickers micro-hardness (Hv) of the B_4_C/Al–Mg–Si composites was performed as per ASTM standard B962-15. The experimental material density was calculated using the Archimedes principle, illustrated in Table 2. Furthermore, to get better outcomes, the tests were performed five times, and the average value of the results was reported.

As the hardness of B_4_C ceramic particles is greater than Al-Mg-Si matrix, the overall hardness of B_4_C modified Al-Mg-Si matrix is close to 98 HV. A higher hardness of the composites is attributed to intrinsic hard characteristics, dispersion strengthening, and good interparticle bonding of boron carbide ceramic particulates in the AMCs [28,29]. The B_4_C particles act like dispersion hardening agents in the matrix and improve the matrix hardening along with the grain size, strengthening of the matrix.

### 3.3. OCP Measurements

**Figure 3:**
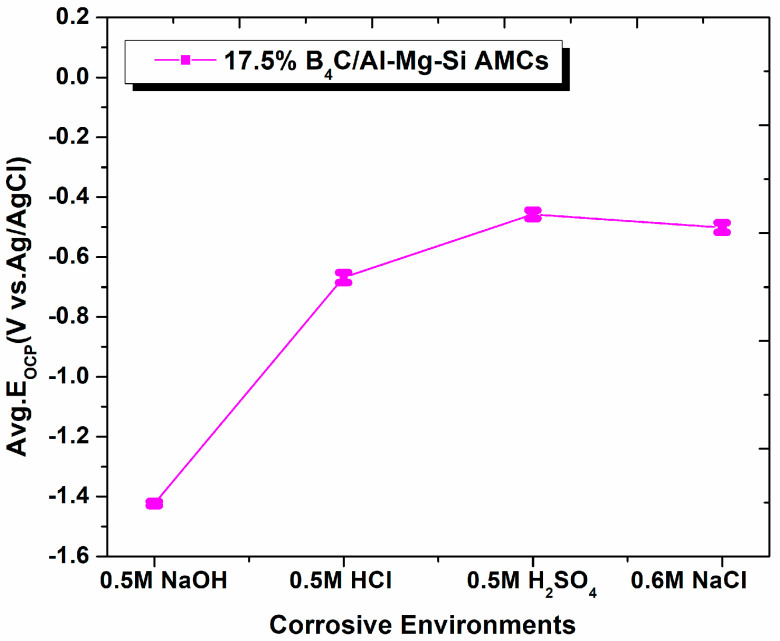
Variations of Average OCP with respect to corrosive environment of 17.5%B_4_C/Al-Mg-Si AMCs.

The OCP vs. corrosive media for the 17.5% B_4_C/Al-Mg-Si AMCs in different environments at 27 ± 0.5 °C are shown in Figure 3. After immersion of AMCs samples into the environments, the OCP values of the samples change positively, as predicted from experiments. As a consequence, it is worthwhile to note that the average OCP of AMCs is seen to be lower in NaCl (OCP_NaCl_ = −0.458 ± 0.050 mV) than NaOH (OCP_NaoH_ = −1.424 ± 0.051 mV) medium. Furthermore, the average OCP appears to be higher in H_2_SO_4_ environments (OCP_H2SO4_ = −0.502 ± 0.050 mV) with respect to HCl (OCP_HCl_ = −0.671 ± 0.050 mV).

It is concluded that after an interval (1 h), the average OCP of AMCs is nobler in NaCl environments. The OCP values for different environments are in the following order of NaOH > HCl > H_2_SO_4_ > NaCl. Similar types of corrosion and thermodynamics stability have been observed due to the incorporation of ceramic reinforcement particles in AMCs [36,37]. The OCP value indicates the stability of the oxide layer formed on the surface of samples. Thus, we can see that the passivation of composites is established at a lower voltage in NaCl as compared to other media.

### 3.4. Potentiodynamic Polarization Plots

**Figure 4:**
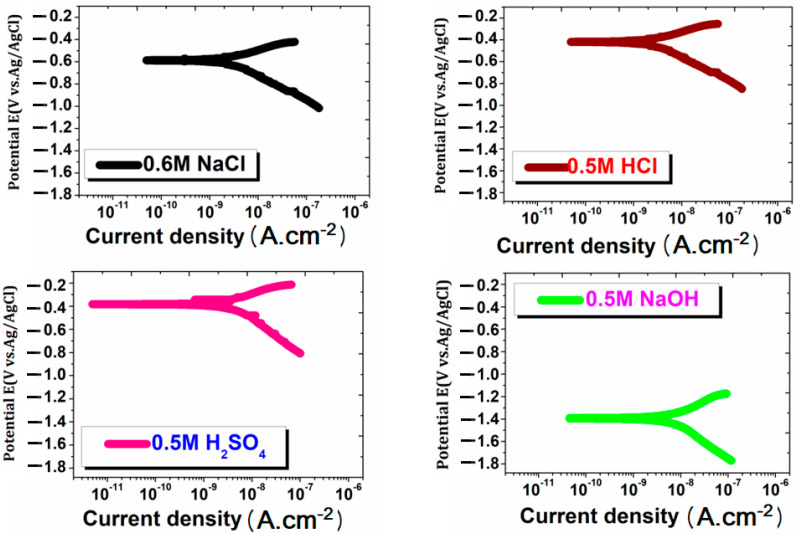
PDP plots of 17.5%B_4_C/Al-Mg-Si AMCs obtained in various environments.

The PDP plots of 17.5% B_4_C/Al-Mg-Si AMCs in various environments at room temperature (27 ± 0.5 °C) are shown in Figure 4. It can be noticed that the corrosion current density (8.3500 ± 0.125 nA/cm^2^) of AMCs sample in 0.6 M NaCl is still in good agreement means higher corrosion resistance than the rest of the environments. In addition, the corrosion current density (16.123 ± 0.005 nA/cm^2^) of AMCs in 0.6 M NaOH revealed less corrosion resistance. If we compare AMCs in the HCl and H_2_SO_4_ solution, then it was shown that corrosion current density (14.401 ± 0.072 nA/cm^2^) in HCl solution seems greater than H_2_SO_4_ solution but smaller than that in NaOH solution, where corrosion current density is 16.123 ± 0.005 nA/cm^2^. The PDP parameters for AMCs, such as corrosion current density (i_corr_), corrosion potential (E_corr_), and open circuit potential (OCP), are further summarized in Table 2.

A lower value of corrosion current density and OCP value indicates the high corrosion resistance. The OCP for NaCl medium is least but the E_corr_ is least for H_2_SO_4_. It can be concluded that the stability of oxide layer is higher in NaCl medium but corrodes in NaCl medium due to the presence of Cl^−^ ions.

### 3.5. Electrochemical Impedance Spectroscopy

**Figure 5:**
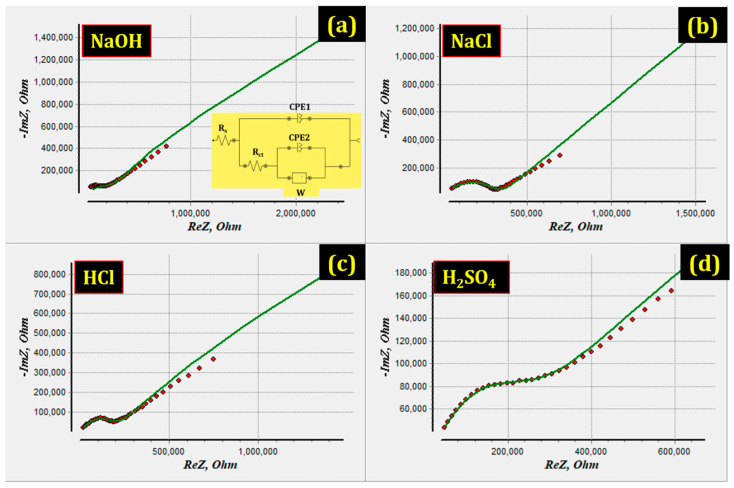
EIS Nyqist plots of 17.5%B4C/Al-Mg-Si AMCs obtained in various environments.

**Figure 6:**
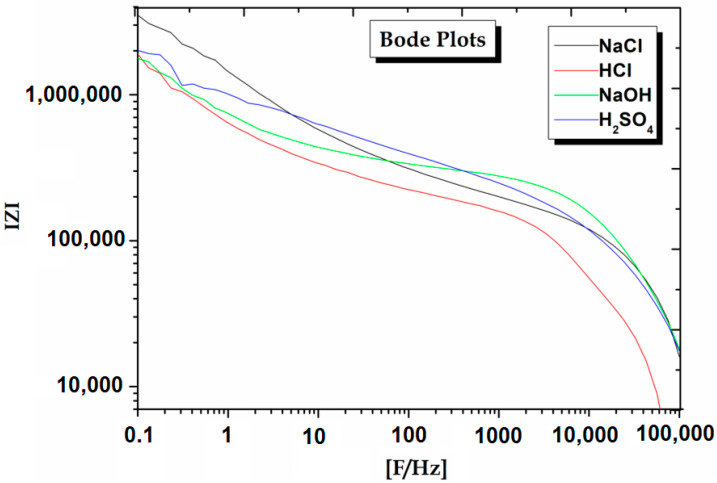
EIS Bode plots of 17.5%B_4_C/Al-Mg-Si AMCs obtained in various environments.

To better understand the experimental outcomes, EIS studies are also undertaken. The Nyquist and Bode plots obtained from EIS are displayed in Figure 5 and Figure 6. The Raddle and Warburg model can be described with the help of EIS data (Nyquist plot). This model contains a charge transfer resistor (R_ct_) and a resistor owing to the Faradaic charge transfer between metallic matrix and environments. The extent R_ct_ in the high-frequency region along the Z’ axis is described by the diameter of the semicircle in the Nyquist plots. The larger the semicircle diameter, the better the corrosion resistance of AMCs in the environments.

Furthermore, the presence of a protective film on the AMCs surface has been confirmed using EIS. As a protective layer is developed on the metallic surface, the charge transfer resistance (R_ct_) grows, the double-layer capacitance value (C_dl_) declines, and the impedance log (Z/Ohm) goes up. The presence of Warburg impedance (W) indicates the diffusion process throughout the corroded films involving electrolyte penetrating into the pores.

The results indicate that R_ct_ increases in NaCl environments meaning higher corrosion resistance as shown by Nyquist and Bode plots. The EIS evidence follows the potentiodynamic polarization (PDP) observations (Table 2). When 17.5%B_4_C/Al-Mg-Si AMCs is immersed in NaCl environments, the R_ct_ value increases from 3.327 × 10^5^ Ohm·cm^2^ to 2.0352 × 10^5^ Ohm·cm^2^ in NaOH medium. CPE_1_ is the capacitance between the solution and the AMCs while CPE_2_ is the interface capacitance at AMC and oxide layer. The CPE_1_ value of NaCl 1.208 × 10^−9^ F·cm^−2^) decreases when compared to that of NaOH (5.182 × 10^−8^ F·cm^−2^). The CPE_2_ values showed no particular trend, which may be due to the change in the oxide structure at the interface. The solution resistance (R_s_) of NaCl also increased to 1.9732 Ohm·cm^2^ as compared to that of NaOH (0.4417 Ohm·cm^2^) environment. These data show that AMCs in NaCl environments can become more stable and its outcome agrees with the PDP results [28,38,39,40,41,42,43,44].

### 3.6. Effect of Temperature on Corrosion Behavior in Cl-Environment

**Figure 7:**
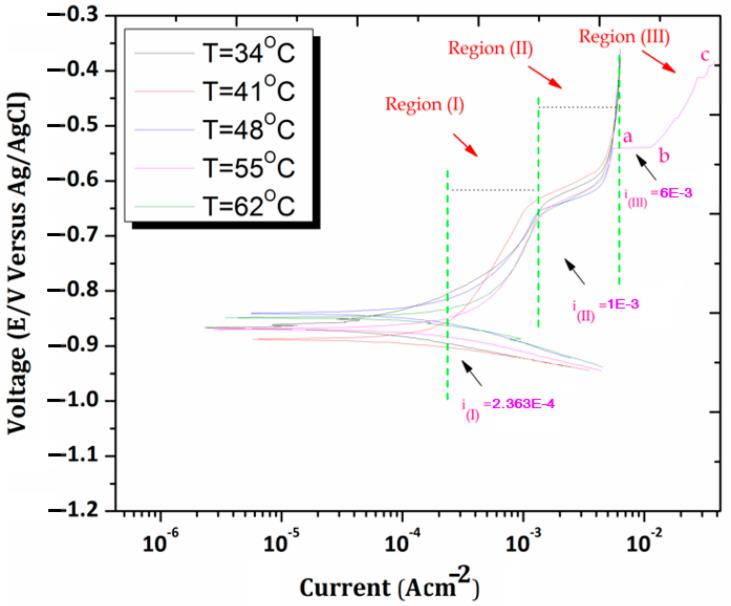
PDP plots of 17.5%B_4_C/Al-Mg-Si AMCs at various temperatures in 0.5 M NaCl environments.

The highest corrosion resistance of the samples investigated in this study was further verified at elevated temperatures in the NaCl medium. The PDP Tafel plots of AMCs are shown at various temperatures between 34 ± 0.5 °C and 62 ± 0.5 °C in NaCl. The graphs portray that the corrosion current density shows a variation with temperature changes. The corrosion potential also shifts to higher negative values with increased temperature of the environment. It means that the corrosion rate increases with increasing temperature. The subsequent reason may be associated with the fact that as one increases the temperature, the oxide film formation over composite becomes thin, spongy, and less defensive by virtue of the dissolution of the film. The plots show slightly different behavior of AMCs at the specific temperature 55 °C. The corrosion current density at point **a** (0.005 A/cm^2^) reaches point **b** (0.0109 A/cm^2^) and it gets stuck at point **c** (0.035 A/cm^2^) in Figure 7.

The succeeding corrosion current value at discussed points at 55 °C showed a very interesting result that corrosion current density increases from initial point **a** to **b** (vertical line). This meant that the metal begins to dissolve in the Cl-environment, which is in the agreement with breakdown protective film formation over a metallic surface. Besides, the Tafel curves display various anodic regions, as stated in the literature for aluminum in NaCl medium.

**Region 1**: Tafel activity region

The corrosion potential, E_corr_, is an active aluminum dissolution region known as the Tafel activity region. Here, current density rises from 2.363 × 10^−4^ to 1.00 × 10^−3^ A/cm^2^ under i(1) to i(II) with influences of potential. This meant that the anodic process used to occur in such regions and there is the formation of soluble species in this domain. The dissolution of Al matrix can occur through the following reactions [45].

Al = Al^3+^ + 3 e^−^ (1)

Al^3+^ + 3Cl^−^ = AlCl_3_ + 3 e^−^ (2)

AlCl_3_ + 3Cl^−^ = AlCl_4_ (3)

It can be traced to two stage process, such as the charge transfer at the electrode to interface and diffusion towards bulk solutions.

**Region 2:** Maximum current density region

In this region, the current density is gradually increasing and has reached its maximum limit. As soon as it reaches the boundary of region III, the current density becomes stagnant. This allows to reveal the existence of a broad passive region in the polarization curve. The presence of this passive region is most likely caused by the formation of the aluminum oxide (Al_2_O_3_) layer over the aluminum composite surface as a result of the reactions mentioned below [46].

O_2_ + 2H_2_O + 4e^−^ = 4OH^−^ (4)

Al + 3OH^−^ = Al(OH)_3_ +3e^−^ (5)

2Al (OH)_3_ = Al_2_O_3_·3H_2_O (6)

The alumina layer further dissolves forming the aluminum hydroxide layer deposits (Al(OH)_3_) according to the reactions (5)–(6).

**Region 3:** Abrupt current region

As the temperature is increasing, the AMCs are being pushed into the passivation region (0.01100–0.01105 A/cm^2^) and significant evidence is illustrated in PDP plots that the i_corr_ shows an abrupt increase (0.006383–0.039400 A/cm^2^) at 55 °C. This indicates the rupture of the passive layer, e.g., Al_2_O_3_·3H_2_O film over the metallic surface as a result of the influence of temperature, and favors the proliferation of corrosion.

### 3.7. Analysis of Corroded Surfaces

**Figure 8:**
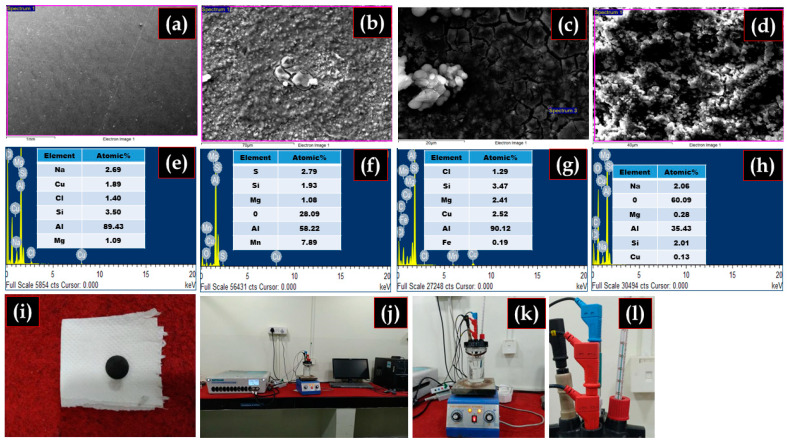
SEM morphology of AMCs after corrosion test (**a**) NaCl (**b**) H_2_SO_4_ (**c**) HCl and (**d**) NaOH, (**e**–**h**) EDS analysis of respective AMCs sample after corrosion, and (**i**–**l**) As fabricated sample and experimental setup for corrosion testing.

The surface morphology of the corroded surfaces and products is examined by SEM-EDS analysis as shown in Figure 8. The severe corrosion attack was found as a cracked layer and deep pits in the environment. The EDS spectrum in Figure 8a reveals that AMCs surfaces are fully rich with Al, Mg, Na, and Cl and there are no other elements. It means that no corrosion product appears to be formed at the composite surface. Further, a corresponding EDS spectrum in Figure 8b predicts the deposition of corrosion products on the composite surface during chemical reactions, and several isotropic cracks on the surface can be seen in the AMCs. The EDS results point to those corrosion products rich in Al, Mg, Na, S-rich compounds. Furthermore, the EDS spectrum in Figure 8c shows the elements, such as Al, Mg, and Cl, and this indicates chloride deposits which further degrade the matrix due to the reaction and evolution of HCl gas. Additionally, the EDS spectrum in Figure 8d indicates a severe effect of corrosion due to enhanced surface cracking.

The spectrum reveals the presence of NaOH, which indicates that a primary oxide film resulting from an OH^-^ ions attack forms more on the AMCs surface. This could be a possibility due to the discontinuities in the layer owing to the dispersed B_4_C ceramic particles in composite samples [47].

## 4. Conclusions

The systematic investigation of the electrochemical corrosion behavior of the 17.5% B_4_C/Al-Mg-Si AMCs in various environments by various electrochemical techniques was performed in this study. PDP and EIS tests of AMCs in various environments revealed that the corrosion resistance of AMCs is greater in the NaCl environments. The SEM morphology of the AMCs showed that the B_4_C ceramic particles were uniformly distributed in the Al-Mg-Si matrix. The experimental findings also indicate that the corrosion resistance decreases with increasing temperature in NaCl environments. The EDS results also indicated that the higher corrosion resistance of AMCs in this study in NaCl is due to the formation of OH^−^ deposits while the unreinforced alloy showed Cl^−^ ion rich deposits. The formation of HCl due to the Cl^−^ reaction further degrades the performance of unreinforced alloy as there is no B_4_C particle barrier that prevents dissolution of the matrix.

## Figures and Tables

**Table 1:** Different corrosive media used for electrochemical corrosion experiment.

Corrosive Environments	Medium	Molarity	pH
Alkaline	NaOH	0.5	8.5
Neutral	NaCl	0.6	6.9
Acidic	H_2_SO_4_	0.5	0.3
Acidic	HCl	0.5	1.0

**Table 2:** PDP and EIS corrosion parameters and mechanical properties of fabricated 17.5% B4C/Al-Mg-Si AMCs in various environments.

PDP Parameters							
Environment	E_corr_ (V)	i_corr_ (nA/cm^2^)	OCP (V)	Mechanical Properties			
NaCl	−0.605 ± 0.20	8.35 ± 0.0001	−0.458 ± 0.016	**Experimental Density (g/cm^3^)**			**Hardness (HV)**
H_2_SO_4_	−0.397 ± 0.11	1.186 ± 0.0014	−0.502 ± 0.014	2.6681 ± 0.021			98.0 ± 2.25
HCl	−0.415 ± 0.05	1.440 ± 0.0205	−0.671 ± 0.017				
NaOH	−1.378 ± 0.11	1.613 ± 0.0003	−1.424 ± 0.007				
**EIS Measurement**							
**Environments**	**Rs, Ω·cm^2^**	**R_ct_ (Ω** **·cm^2^)**	**CPE_1_**		**CPE_2_**		**W (Ohm·cm^2^)**
			**p_1_ (F·cm^−2^)**	**n_1_**	**p_2_ (F·cm^−2^)**	**n_2_**	
NaCl	1.9732	3.3271 × 10^5^	1.208 × 10^−9^	0.653	1.851 × 10^−7^	0.423	1.20 × 10^6^
H_2_SO_4_	1.8551	2.7787 × 10^5^	1.870 × 10^−9^	0.642	2.343 × 10^−7^	0.362	3.62 × 10^6^
HCl	1.8241	2.0610 × 10^5^	2.150 × 10^−9^	0.676	2.603 × 10^−7^	0.412	6.69 × 10^6^
NaOH	0.4417	2.0352 × 10^5^	5.182 × 10^−8^	0.613	8.159 × 10^−7^	0.342	9.57 × 10^6^

## Data Availability

The data relating to this work are confidential and cannot be shared at this time.
